# A Novel Hybrid Deep Learning Method for Fault Diagnosis of Rotating Machinery Based on Extended WDCNN and Long Short-Term Memory

**DOI:** 10.3390/s21196614

**Published:** 2021-10-04

**Authors:** Yangde Gao, Cheol Hong Kim, Jong-Myon Kim

**Affiliations:** 1 Department of Electrical, Electronics and Computer Engineering, University of Ulsan, Ulsan 44610, Korea; gaoyangdephd@gmail.com; 2 School of Computer Science and Engineering, Soongsil University, Seoul 06978, Korea; cheolhong@ssu.ac.kr

**Keywords:** deep learning, fault diagnosis, rotating machinery, Extended Deep Convolutional Neural Networks, long short-term memory

## Abstract

Deep learning (DL) plays a very important role in the fault diagnosis of rotating machinery. To enhance the self-learning capacity and improve the intelligent diagnosis accuracy of DL for rotating machinery, a novel hybrid deep learning method (NHDLM) based on Extended Deep Convolutional Neural Networks with Wide First-layer Kernels (EWDCNN) and long short-term memory (LSTM) is proposed for complex environments. First, the EWDCNN method is presented by extending the convolution layer of WDCNN, which can further improve automatic feature extraction. The LSTM then changes the geometric architecture of the EWDCNN to produce a novel hybrid method (NHDLM), which further improves the performance for feature classification. Compared with CNN, WDCNN, and EWDCNN, the proposed NHDLM method has the greatest performance and identification accuracy for the fault diagnosis of rotating machinery.

## 1. Introduction

In modern industries, rotating machinery is widely used in complex mechanical fields that work under severe conditions. Therefore, various failures can occur over a long running time. Fault diagnosis uses various methods to ensure safe operation and reduce losses for rotating machinery, which is very important [1,2]. Traditionally, fault diagnosis contains signal acquisition, feature extraction, and fault classification that can detect most intrinsic mechanical information and identify fault features [3,4,5]. There are a variety of fault diagnosis methods, including time-frequency analysis, that can analyze the characteristic frequency of a vibration signal in the time domain; however, more time is required for data consumption. A wavelet transform (WT) was presented to filter noise and analysis features for vibration signals. Even so, WT still suffers some drawbacks when processing large amounts of nonlinear vibration signals [6]. Empirical Mode Decomposition (EMD) was successfully applied to the fault diagnosis of the vibration signal; however, after extracting impulsive features from the vibration signals, the mode mixing problem remained [7].

In recent years, many methods based on machine learning have been developed for fault diagnosis. Support vector machine (SVM) has an advantage as a global optimal solution that can be widely applied to classification in fault diagnosis; however, SVM is a shallow model and cannot immediately achieve feature self-learning from vibration signals [8,9]. The k-nearest neighbor (KNN) method can achieve a good performance for classification; however, it still has some difficulties for high-dimensional data [10]. Machine learning methods have achieved good performances for classification of rotating machinery. However, their architecture still lacks multi-layer nonlinear mapping ability, and as a result they cannot fully use previous information for classification, and existing methods need to exhibit better performances for the amount of data in complex conditions [11,12,13,14].

Neural networks have the advantage of self-learning for fault prediction, and classification ELM has a simple structure and can achieve a fast learning speed for higher prediction performance. In addition, Artificial Neural Networks (ANNs) are used for the fault diagnosis of vibration signals and the classification performance relies on the feature quality of vibration signals [15]. However, these methods still have inherent shortcomings. The parameters of similar approaches should be designed by humans before classifying features for vibration signals, and their effectiveness cannot be guaranteed in various optimal feature extractions. Additionally, when processing a massive dataset, they do not have very strong self-learning capability for complex classification.

Compared with traditional machine learning methods, deep learning (DL) can tackle multiple nonlinear processing layers, so it can automatically achieve self-learning and feature detection, which is very popular in many research fields. DL is based on modularized automatically learning network architectures; similarly, convolutional neural networks (CNN) are used to capture impact features of vibration signals [8]. Densely connected CNNs (DCNNs) are introduced to reinforce the collection learning of vibration features due to the weight reuse and better performance than traditional CNN [12]. The WDCNN model can achieve a better performance for extraction features; however, when considering a complex condition, it still requires a large amount of data for training and decreases rapidly; although complex DL models can achieve good results for diagnosis, they still consume a lot of computational time and training examples [2,10]. Therefore, to solve these problems, it is necessary to develop a novel hybrid DL method to improve the diagnosis performance of vibration signals.

Long short-term memory (LSTM) uses architecture to address the gradient vanishing and exploding problem, which can be used for prediction [16,17]. LSTM can also helps CNN develop the CNN-LSTM, which has stronger diagnostic ability. The CNN-LSTM network is constructed to analyze the vibration signals; when the CNN method finishes the feature of one-dimensional singles, LSTM continues to process this important information for diagnosis classification [18,19,20]. LSTM can extract special correlations for the stronger self-learning ability of CNN for prediction, and these architectures produce many ideas that are useful for this paper [21,22,23].

To address the above problems, the improved WDCNN method was combined with the LSTM method to produce a novel hybrid method for fault classification of rotating machinery. The proposed model uses the reasonable network structure and depth fusion of each neural network, and in each DL iteration, the model uses previous feedback and LSTM information to optimize extraction features and decrease the errors for fault classification. The contributions of this paper are:

(1) The novel hybrid DL method can be implemented under different bearing health conditions, as it fully uses previous information for extraction features and has a stronger self-learning capacity to achieve a high accuracy for fault classification.
(2) The proposed method integrates the advantages of the improved WDCNN method and LSTM method, and uses various neural networks for stronger features of self-learning from large vibration signals.
(3) The t-distributed Stochastic Neighbor Embedding (t-SNE) can show different mapping abilities of the neural networks for fault classification. This visualization can display how different layers capture the information step by step, and ten different fault styles of vibration bearing signals were easily recognized by the novel hybrid DL method.

The rest of the paper is organized as follows. In Section 2 and Section 3 the basic theory of the WDCNN method and LSTM method are introduced, respectively. In Section 4, the improved WDCNN method and proposed framework of the novel hybrid deep learning method (NHDLM) are described in detail. In Section 5, experiments are presented to demonstrate the different performances of the traditional CNN method, WDCNN method, EWDCNN method, and the proposed method (NHDLM). The conclusions are presented in Section 6.

## 2. Architecture of the WDCNN Model

To address the problems of the traditional CNN method, the WDCNN method was proposed for 1-D vibration signals under different conditions. The overall architecture contains convolutional layers, pooling layers, and one classification stage. Compared with the traditional CNN method, this architecture takes advantage of the first wide kernels to capture useful information from high-frequency noise signals, and then small kernels were used to acquire low frequency features. The multilayer convolutional kernels make the networks deeper.

### 2.1. Convolutional Layer

In the convolutional layer, convolution operation was conducted on the input local region with filter kernels/weight and the output features generated from input signals, input $x^{l}\left(j\right)$, convolution kernel $K_{i}^{l},\;$ and bias $b_{i}^{l}\;$ of the i− th filter kernel in layer l to produce an output feature map of the j− th local region in layer l. The convolution process is described as follows:

(1) $$y_{i}^{l+1}(j)\;=\;K_{i}^{l}*x^{l}(j)\;+\;b_{i}^{l}$$

where the * notation computes the dot product of the kernel and local regions. 

### 2.2. Activation Layer

After the convolution operation, a Rectified Linear Unit (ReLU) was used to enhance the representation ability and learn features for convolutional networks. As a useful activation unit, ReLU can adjust the parameters for training layers by weights, where $y_{i}^{l+1}\left(j\right)$ is the output value of the convolution operation. The formula is described as follows:

(2) $$a_{i}^{l+1}(j)\;=\;f(y_{i}^{l+1}(j))\;=\;max\left\{0,y_{i}^{l+1}(j)\right\}$$

where $a_{i}^{l+1}\left(j\right)$ is the activation.

### 2.3. Pooling Layer

After the convolutional layer in the architecture, a max-pooling layer was used to reduce the spatial size of the features and parameters of a neuron in the previous layer, which performs the local max operation over the input feature map $x_{i}$ and produces the output feature map $y_{i\;}$. The max-pooling layer is described as follows:

(3) $$y_{i}\;=\;max(x_{i})$$

where the formula is a pooling operation for max-pooling and $y_{i}$ denotes the corresponding value of the neuron.

### 2.4. Batch Normalization

After a convolutional layer or fully connected layer, the batch normalization (BN) layer was applied to reduce the shift of internal covariance. When input datum is $x\;=\;\left(x^{1},\cdots ,x^{p}\right)$, the BN layer is described as:

(4) $$\begin{array}{c} {\hat{x}}^{i}=\frac{x^{i}-E\left[x^{i}\right]}{\sqrt{Var\left[x^{i}\right]}}\\ y^{i}=\gamma^{i}{\hat{x}}^{i}+\beta^{i} \end{array}$$

where $\gamma^{i}$ is the scale parameter, $\beta^{i}$ is shift parameter, and $y^{i}$ is the output.

### 2.5. Architecture of the WDCNN Model

**Figure 1:**
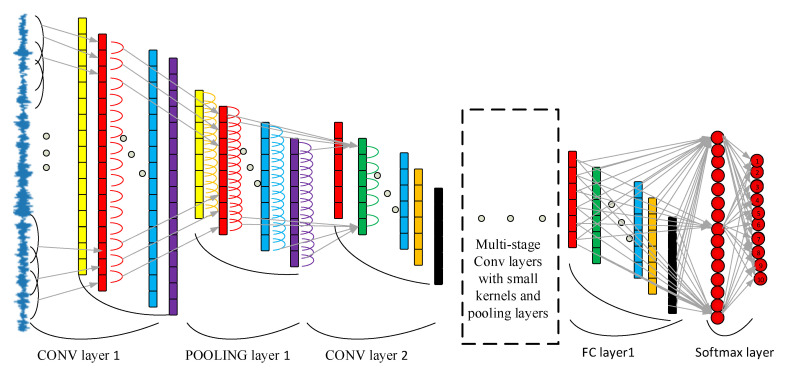
Architecture of the WDCNN model.

The overall architecture of WDCNN has filter stages and a classification stage as a traditional CNN model, as shown in Figure 1. However, the WDCNN model takes advantage of the first wide convolutional layer and multi-stage convolutional layers for stronger extraction features for input vibration signals. The WDCNN is used for 1-D input vibration signals without any other transformation, and multi-layer small convolutional kernels can make the networks deeper to extract good representation. Batch normalization and a fully connected layer are then used to accelerate the training process.

After extracting the features, the classification stage uses the fully connected layers for fault style classification. For the output layer, the SoftMax function used logits of ten neurons for the probability distribution of ten different bearing health conditions, described as:

(5) $$q\left(z_{j}\right)\;=\;\frac{e^{z\_j}}{\sum_{k}^{10}e^{z\_j}}$$

where $z_{j\;}$ is the logits of the j−th output neuron.

## 3. Long Short-Term Memory (LSTM)

**Figure 2:**
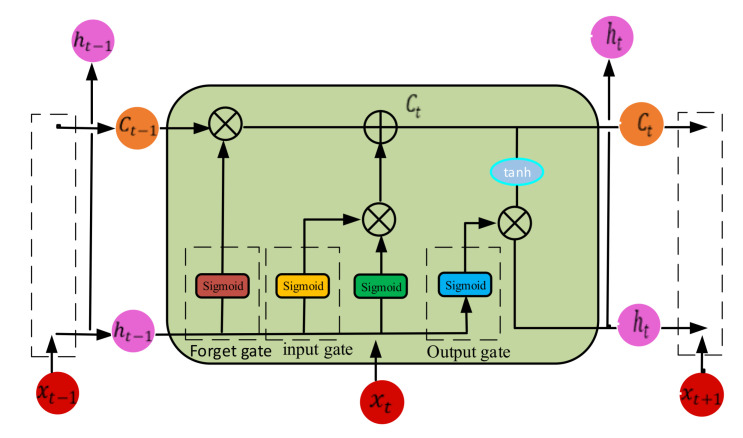
The unfolded structure of LSTM.

As a modified version of a recurrent neural network (RNN), LSTM can take advantage of current and previous information of the current task, and can address the drawback of traditional RNN for long-term memory. The LSTM network contains four gates, the forget gate, update gate, input gate, and output gate. The LSTM method, as shown in Figure 2, can flexibly keep the long-term memory of previous learning information, which means that the architecture of LSTM is more suitable for processing time series than RNNs.

The forget gate was applied to capture important features from the previous neuron state in a one-layer neural network. The forget gate is described as follows:

(6) $$f_{t}=sigmoid(W_{f}^{T}h_{t-1}+U_{f}^{T}x_{t}+b_{f})$$

where $x_{t}$ is the input vector at time t, $h_{t-1}$ is the output of the memory block at time *t* − 1, $W_{f}^{T}$, $U_{f}^{T}$ are the weight vectors, and $b_{f}\;$ is the bias vector. The input gate $i_{t}\;$ can determine how much new information should be added to the current state of the neuron:

(7) $$i_{t}=sigmoid\left(W_{i}^{T}h_{t-1}+U_{i}^{T}x_{t}+b_{f}\right)$$

The new memory content is calculated and updated as follows:

(8) $$\tilde{C_{t}}=tanh\;\left(W_{c}^{T}h_{t-1}+U_{c}^{T}x_{t}+b_{c}\right)$$

(9) $$C_{t}=f_{t}*C_{t-1}+i_{t}*\tilde{C_{t}}$$

where $C_{t}$ is the state information, $\tilde{C_{t}\;}$ is the candidate state information, and tanh is the hyperbolic tangent activation function. The output gate $o_{t}$ is calculated to determine how much information should be used in the next time step:

(10) $$o_{t}=sigmoid\;\left(W_{o}^{T}h_{t-1}+U_{o}^{T}x_{t}+b_{o}\right)$$

The output of memory block is calculated as follows:

(11) $$h_{t}=o_{t}tanh\;\left(C_{t}\right)$$

In the architecture of LSTM, the memory unit $C_{t}$ depends on the forget gate output $f_{t}$, the candidate memory information $\tilde{C_{t}}$, and the long-term memory information $C_{t-1}$. The output $h_{t}\;$ of memory block depends on output $o_{t}$ of the output gate and value the memory unit $C_{t}$.

## 4. The Proposed Novel Hybrid Deep Learning Method

**Figure 3:**
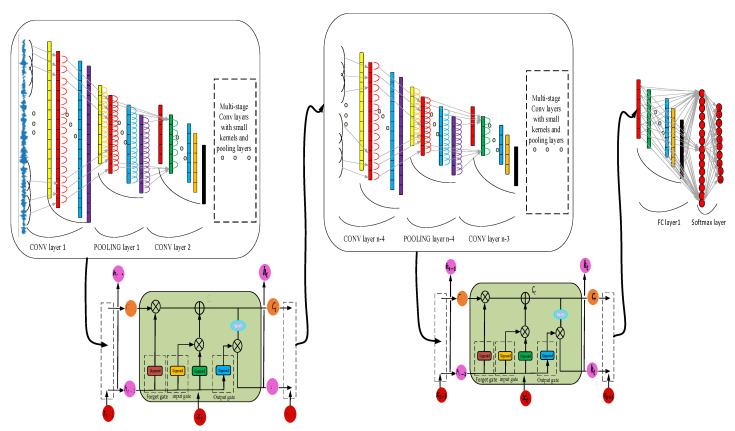
Architecture of the proposed NHDLM model.

Before the proposed NHDLM method, the EWDCNN method was developed by extending the convolution layer of WDCNN. The convolutional layers were added to the architecture to develop the EWDCNN. Compared with the WDCNN model, the EWDCNN has six convolutional layers and results in a stronger deep learning capacity for signals. The architecture of EWDCNN was then exchanged with LSTM and the novel hybrid deep learning method (NHDLM) was developed, as shown in Figure 3. The architecture mainly consists of six convolutional layers with pooling layers, two LSTM, and one full connection layer.

**Figure 4:**
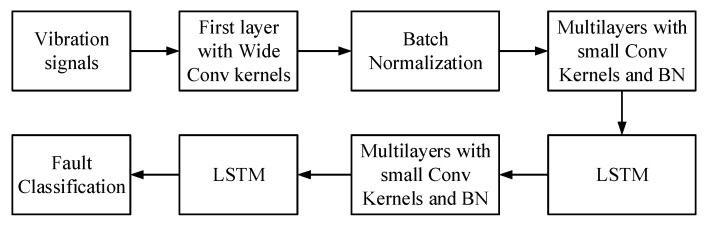
The Fault Classification framework of the proposed NHDLM method.

The novel hybrid deep learning method is designed to extract the spatial and temporal variation features of 1-D vibration bearing signals. The multiple convolutional layers can remove the noise from the vibration signals and extract the special features step by step. The proposed method based on two LSTMs can also take advantage of keeping the long short-term memory for extracting the time variation features of vibration signals and improve prediction. Finally, the full connection layer was used to integrate the features information, which was convenient for fault classification, realizing the nonlinear mapping, as shown in Figure 4.

## 5. Experiment

**Figure 5:**
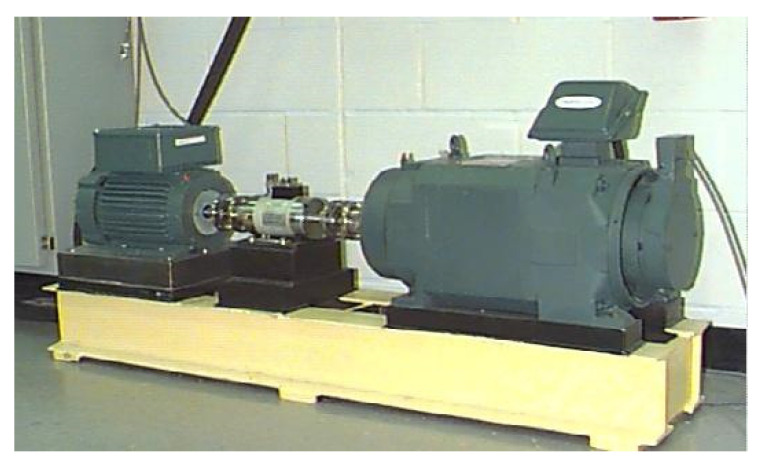
Motor driving mechanical system used by CWRU.

To validate this proposed method, public experimental data from Case Western Reserve University (CWRU) were applied for fault classification, as shown in Figure 5. This experiment platform includes a transducer, dynamometer, and induction motor. The testing sampling frequency was 12 kHz, and in addition to the Normal Condition (NC), there were also three fault types of the vibration bearing: Ball Fault (BF), Inner Race Fault (IF), and Out Race Fault (OF). Each fault type has three levels of severity with fault diameters of 0.07 inches, 0.014 inches and 0.021 inches, respectively. There were ten data styles of bearing health for training and testing: NC, BF7, BF14, BF21, IF7, IF14, IF21, OF7, OF14, and OF21. Each sample has 1024 points. Datasets A, B, C, and D, respectively, contain 700 training samples and 100 testing samples, 1400 training samples and 200 testing samples, 2100 training samples and 300 testing samples, and 2800 training samples and 400 testing samples of ten different fault conditions under loads of 0, 1, 2, and 3 hp. More details of the datasets are described in Table 1.

**Figure 6:**
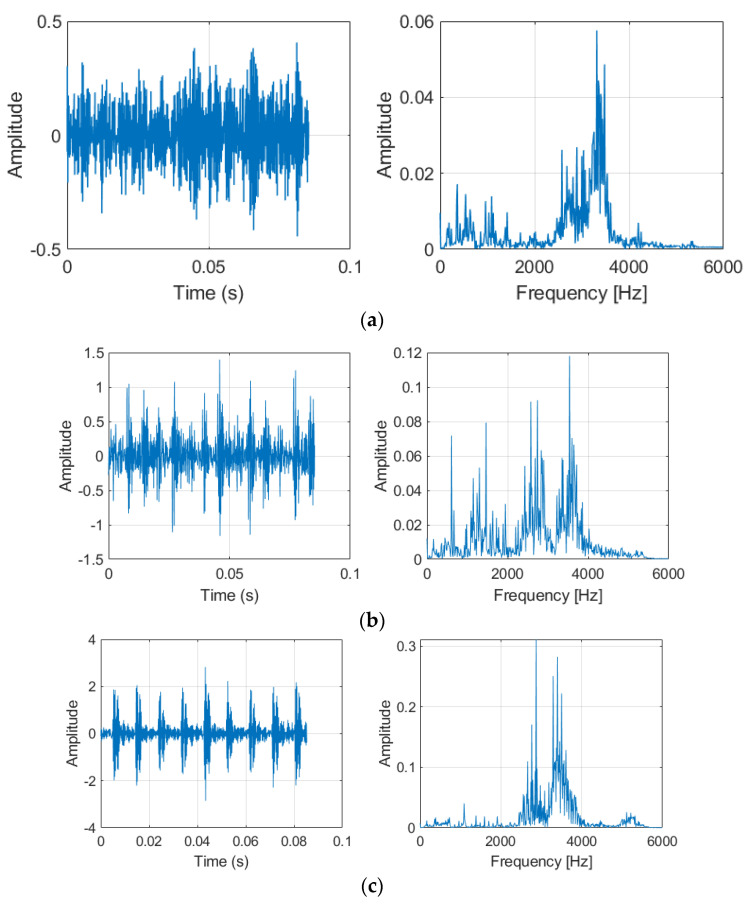
Vibration signatures of bearing fault. (**a**) Ball Fault (BF) and Frequency. (**b**) Inner Race Fault (IF) and Frequency. (**c**) Out Race Fault (OF) and Frequency.

We also add vibration signatures of bearing fault in Figure 6. It is very difficult to diagnose for Ball Fault (BF) as many signals have impulsive content, and the ball fault is only engaging with the races in signals and seemingly at random. The Inner Race Fault (IF) exhibits some strong harmonics, with very clear impulsive modulation at shaft speed. Out Race Fault (OF) exhibits unnormal characteristic symptoms in the envelope spectra and has the most modulation at shaft speed.

### 5.1. Parameters of the Proposed Novel Hybrid Deep Learning Method

In this experiment, the architecture of the proposed novel hybrid DL method has 6 convolutional and pooling layers, 2 LSTM networks, fully connected hidden layers, and a soft-max layer. In this architecture, the size of the first convolutional kernel is 64 × 1 and the rest of the kernel sizes are 3 × 1. Max pooling is added after each convolutional layer, and then batch normalization is used to improve the performance of the method to select the adaptive size of the neuron. The parameters of the convolutional layers and LSTM network are detailed in Table 2, where Google TensorFlow and Python 3.7 were applied for the experiment. 

### 5.2. Feature Visualization Analysis

**Figure 7:**
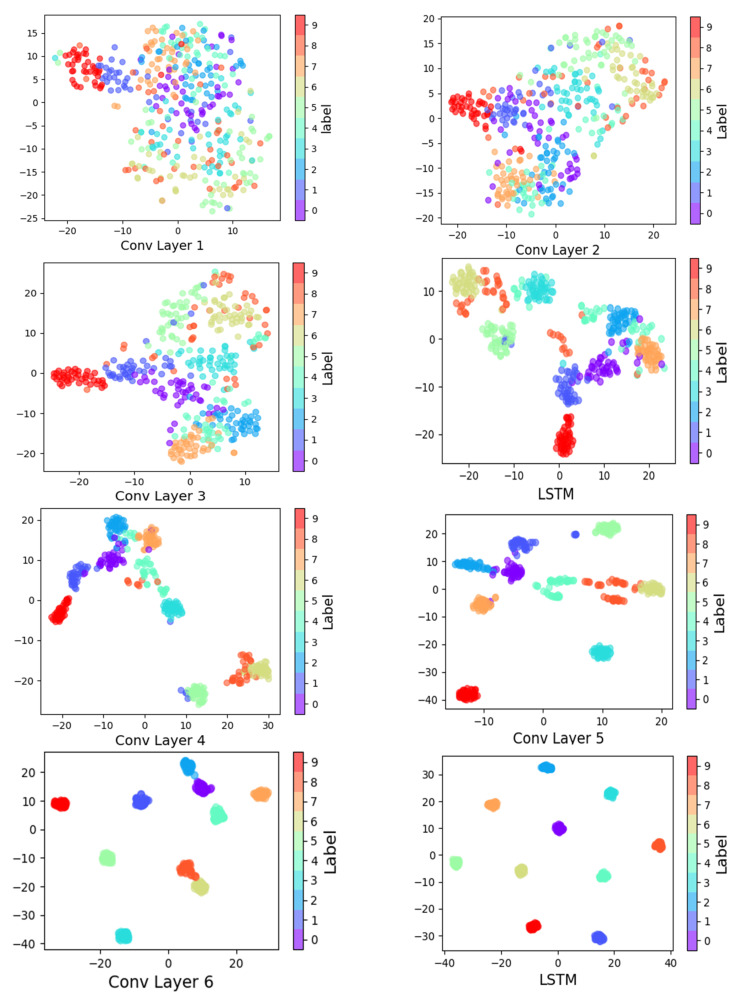
Feature visualization via t-SNE: feature representation for ten fault styles of vibration signals extracted from 6 convolutional layers and 2 LSTM networks.

According to feature visualization by mapping, the t-distributed Stochastic Neighbor Embedding (t-SNE) was applied to verify the self-learning feature capacity of the novel hybrid deep learning method under different neuron layers, as shown in Figure 7, which means ten fault styles of the vibration signals were easier to recognize. This visualization displays how different layers can capture information step by step using nonlinear mapping. All of the fault signals become separable, and it does not perform very well in early layers; however, as the layer goes deeper, the model can make full use of the self-learning capability for fault classification. The final layer finishes the cluster for ten fault styles of vibration bearing signals.

**Figure 8:**
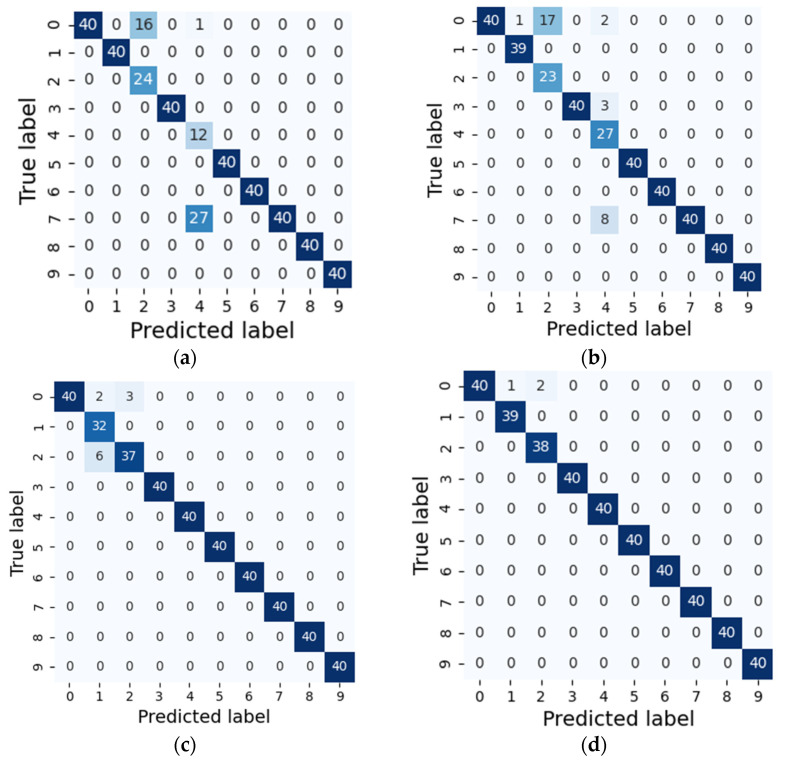
Confusion matrices of different models. (**a**) CNN. (**b**) WDCNN. (**c**) EWDCNN. (**d**) Proposed method.

As shown in Figure 8, there are approximately four confusion matrices for four predictive models (traditional CNN method, WDCNN method, improved EWDCNN method, proposed NHDLM method) trained on the 2800/400 train–test split. In each confusion, a blue rectangle means that all ten fault signals were correctly classified, a green rectangle means that the fault type was not classified correctly, and the number in a rectangle denotes the number of tests. For the first case (predictive model 1-traditional CNN), (400-43) out of 400 tests were correctly classified, which means that the traditional CNN hit an accuracy of 89% on sample testing. For predictive model 2 (WDCNN), (400-31) out of 400 tests were correctly classified for an accuracy of 92%. For predictive model 3 (EWDCNN), (400-11) out of 400 were correctly classified for a prediction accuracy of 97%. For the proposed method, (400-3) out of 400 were correctly classified for a classification accuracy hit of 99%. Many fault types were classified.

**Figure 9:**
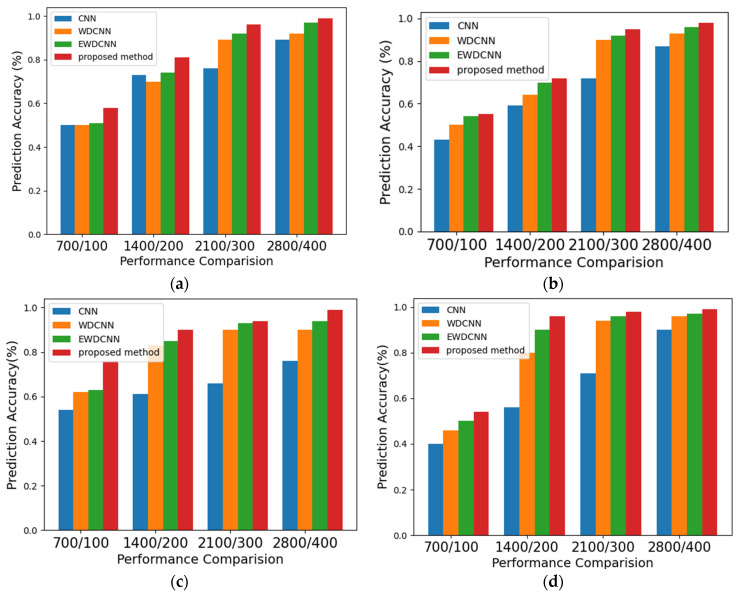
Evaluation indexes of different models. (**a**) Data A. (**b**) Data B. (**c**) Data C. (**d**) Data D.

To further verify the effectiveness of the proposed method in vibration signals, the A, B, C, and D datasets were applied to compare the CNN, WDCNN, and EWDCNN methods with the proposed NHDLM method. The simulation results are presented in Figure 9 and described as below.

(a) Dataset A: For 700 training samples and 100 testing samples, the CNN approach achieved 50% prediction accuracy, the WDCNN method was 50%, the EWDCNN method was 51%, and the proposed NHDLM method was 58%. For 1400 training samples and 200 testing samples, the CNN approach achieved 73% prediction accuracy, the WDCNN method was 70%, the EWDCNN method was 71%, and the proposed NHDLM method was 81%. For 2100 training samples and 300 testing samples, the CNN approach achieved 76% prediction accuracy, the WDCNN method was 89%, the EWDCNN method was 92%, and the proposed NHDLM method was 96%. For 2800 training samples and 400 testing samples, the CNN approach achieved 89% prediction accuracy, the WDCNN method was 92%, the EWDCNN method was 97%, and the proposed NHDLM method was 99%.
(b) Dataset B: For 700 training samples and 100 testing samples, the CNN approach achieved 43% prediction accuracy, the WDCNN method was 50%, the EWDCNN method was 54%, and the proposed NHDLM method was 55%. For 1400 training samples and 200 testing samples, the CNN approach achieved 59% prediction accuracy, the WDCNN method was 64%, the EWDCNN method was 70%, and the proposed NHDLM method was 72%. For 2100 training samples and 300 testing samples, the CNN approach achieved 72% prediction accuracy, the WDCNN method was 92%, the EWDCNN method was 94%, and the proposed NHDLM method was 95%. For 2800 training samples and 400 testing samples, the CNN approach achieved 87% prediction accuracy, the WDCNN method was 94%, the EWDCNN method was 96%, and the proposed NHDLM method was 98%.
(c) Dataset C: For 700 training samples and 100 testing samples, the CNN approach achieved 54% prediction accuracy, the WDCNN method was 62%, the EWDCNN method was 63%, and the proposed NHDLM method was 76%. For 1400 training samples and 200 testing samples, the CNN approach achieved 61% prediction accuracy, the WDCNN method was 83%, the EWDCNN method was 85%, and the proposed NHDLM method was 90%. For 2100 training samples and 300 testing samples, the CNN approach achieved 66% prediction accuracy, the WDCNN method was 90%, the EWDCNN method was 93%, and the proposed NHDLM method was 94%. For 2800 training samples and 400 testing samples, the CNN approach achieved 87% prediction accuracy, the WDCNN method was 94%, the EWDCNN method was 97%, and the proposed NHDLM method was 99%.
(d) Dataset D: For 700 training samples and 100 testing samples, the CNN approach achieved 40% prediction accuracy, the WDCNN method was 46%, the EWDCNN method was 50%, and the proposed NHDLM method was 54%. For 1400 training samples and 200 testing samples, the CNN approach achieved 56% prediction accuracy, the WDCNN method was 80%, the EWDCNN method was 90%, and the proposed NHDLM method was 96%. For 2100 training samples and 300 testing samples, the CNN approach achieved 71% prediction accuracy, the WDCNN method was 94%, the EWDCNN method was 96% and the proposed NHDLM method was 98%. For 2800 training samples and 400 testing samples, the CNN approach achieved 90%, the WDCNN method was 96%, the EWDCNN method was 97%, and the proposed NHDLM method was 99%.

The traditional CNN method still has some drawbacks for larger datasets; based on CNN, the WDCNN method can take advantage of the first wide convolutional layer and multi-stage convolutional layers for stronger extraction features. To enhance the self-learning capacity of WDCNN method, the EWDCNN method is presented by extending the convolution layer of WDCNN, which can further improve automatic feature extraction. The LSTM then changes the geometric architecture of the EWDCNN to produce a novel hybrid method (NHDLM), which further improves the performance for feature classification. So, the proposed NHDLM method had the greatest identification accuracy for bearing datasets.

More information is shown in Table 3 to further illustrate the feasibility of the proposed method for different training and testing.

When the training samples increased from 700 to 2800 and the testing sample increased from 100 to 400, the accuracy of these methods increased very obviously with the increasing samples. With 2800 training samples, the accuracy of the proposed method was greater than 99% and greater than the other methods. The proposed method had the greatest recognition accuracy in different datasets and methods, indicating promise for feature self-learning performance for vibration signals.

To further verify the effectiveness of the proposed method in training time, some datasets were applied to compare the CNN, WDCNN, and EWDCNN methods with the proposed NHDLM method. Training times are presented in Table 4. For 2800 training samples and 400 testing samples, the existing CNN, WDCNN, EWDCNN and the proposed NHDLM require 14.595, 23.311, 22.166, and 30.636 s, respectively.

We also calculate some SNRs of training signals and testing signals, Ball Fault (BF): SNR = −2.3; Inner Race Fault (IF): SNR = −5.8; Out Race Fault (OF): SNR = −4.8; according to the datasets, experiment can prove that the prediction accuracy can show the robustness of the proposed method.

## 6. Conclusions

In this paper, a novel hybrid DL method was proposed for fault classification for rotating machinery under complex working conditions. The health states contain different pressure load, speed, Fault Types, Fault Diameters, and training/testing samples. The proposed method is suitable for processing large datasets about vibration bearing signals and can achieve a good performance for prediction.

Based on the WDCNN method, the EWDCNN method was developed by extending the convolution layer, and LSTM changed the EWDCNN’s geometric architecture to develop the proposed NHDLM method. In the proposed NHDLM method, the architecture has different convolutional layers and LSTM networks. The LSTM networks can effectively increase the self-learning capability of the convolutional layers for ten fault styles of vibration signals, and the proposed model can effectively integrate the layer. The experiment proves that the proposed NHDLM method has a better performance than the existing CNN, WDCNN and EWDCNN methods, and NHDLM can achieve a greater prediction accuracy on different fault styles of vibration signals.

The experiment proved that the proposed NHDLM method exhibits a better performance than the existing CNN, WDCNN, and EWDCNN methods, and that NHDLM can achieve greater prediction accuracy on different fault styles of vibration signals. Before deep learning method process datasets, denoising preprocessing methods are not used. Therefore, some denoising methods can be used to preprocess and improve the performance of the deep learning methods in the future.

## Figures and Tables

**Table 1:** Datasets for vibration bearing fault diagnosis.

Load (hp)	Fault Types	Fault Diameters	Training/Testing
0, 1, 2, 3	NC	0	700/100, 1400/200, 2100/300, 2800/400
0, 1, 2, 3	BF	7	700/100, 1400/200, 2100/300, 2800/400
0, 1, 2, 3	BF	14	700/100, 1400/200, 2100/300, 2800/400
0, 1, 2, 3	BF	21	700/100, 1400/200, 2100/300, 2800/400
0, 1, 2, 3	IF	7	700/100, 1400/200, 2100/300, 2800/400
0, 1, 2, 3	IF	14	700/100, 1400/200, 2100/300, 2800/400
0, 1, 2, 3	IF	21	700/100, 1400/200, 2100/300, 2800/400
0, 1, 2, 3	OF	7	700/100, 1400/200, 2100/300, 2800/400
0, 1, 2, 3	OF	14	700/100, 1400/200, 2100/300, 2800/400
0, 1, 2, 3	OF	21	700/100, 1400/200, 2100/300, 2800/400

**Table 2:** Details of the proposed novel hybrid DL method used in the experiment.

No.	Layer Type	Kernel Size/Stride	Kernel Number
1	Convolution1	64 × 1	16
2	Pooling1	2 × 1	16
3	Convolution2	3 × 1	32
4	Pooling2	2 × 1	32
5	Convolution3	3 × 1	64
6	Pooling3	2 × 1	64
7	LSTM	units = 16	
8	Convolution4	3 × 1	64
9	Pooling4	2 × 1	64
10	Convolution5	3 × 1	64
11	Pooling5	2 × 1	64
12	Convolution6	3 × 1	64
13	Pooling6	2 × 1	64
14	LSTM	units = 16	
15	Fully connected	100	1
16	Softmax	10	1

**Table 3:** Prediction precision for different models.

Data Set	Training/Testing	CNN	WDCNN	EWDCNN	Proposed Method
	700/100	0.5	0.5	0.51	0.58
A	1400/200	0.73	0.7	0.71	0.81
	2100/300	0.76	0.89	0.92	0.96
	2800/400	0.89	0.92	0.97	0.99
	700/100	0.43	0.5	0.54	0.55
B	1400/200	0.59	0.64	0.7	0.72
	2100/300	0.72	0.92	0.94	0.95
	2800/400	0.87	0.94	0.96	0.98
	700/100	0.54	0.62	0.63	0.76
C	1400/200	0.61	0.83	0.85	0.9
	2100/300	0.66	0.9	0.93	0.94
	2800/400	0.87	0.94	0.97	0.99
	700/100	0.4	0.46	0.5	0.54
D	1400/200	0.56	0.8	0.9	0.96
	2100/300	0.71	0.94	0.96	0.98
	2800/400	0.9	0.96	0.97	0.99

**Table 4:** The classification accuracy and time consumption.

Method	Time Consumption	Overall Classification
(Training Size/2800 Samples)	(Second)	Result (%)
CNN	14.595	87%
WDCNN	23.311	94%
EWDCNN	22.166	96%
NHDLM	30.636	98%

## Data Availability

The data are publicly available.
